# Best of Both Worlds: Bridging Research and Practice to Achieve Health Equity

**DOI:** 10.1093/geroni/igaa057.3204

**Published:** 2020-12-16

**Authors:** Karen Lincoln

**Affiliations:** University of Southern California, Los Angeles, California, United States

## Abstract

Health inequity is linked to societal and social determinants that constrain health promoting opportunities for older adults. From birth to death, many older adults contend with the prevailing social and health effects of discrimination in employment, education, and housing. As a consequence, middle-aged Black Americans are experiencing accelerated aging, and living with and dying from preventable chronic health conditions typically diagnosed at older ages. Despite these conditions, there is much variation in health outcomes among older Black Americans. Professor Karen Lincoln will share her approach to research that explores heterogeneity within the Black American population to discover factors that promote healthy aging. She will describe her unique outreach and health education program that serves older adults in underserved communities, and her roles as an aging advocate and public scholar. She will discuss how these experiences led to the development and testing of an innovative intervention to reduce Alzheimer’s disease disparities.

